# An intelligent Bayesian hybrid approach to help autism diagnosis

**DOI:** 10.1007/s00500-021-05877-0

**Published:** 2021-05-24

**Authors:** Paulo Vitor de Campos Souza, Augusto Junio Guimaraes, Vanessa Souza Araujo, Edwin Lughofer

**Affiliations:** 1 Department of Knowledge Based Mathematical Systems, Johannes Kepler University, Linz, Austria; 2 Faculty Una of Betim, Betim, Brazil

**Keywords:** Bayesian fuzzy neural network, Autistic spectrum disorder, Autism prediction, Bayesian fuzzy clustering

## Abstract

This paper proposes a Bayesian hybrid approach based on neural networks and fuzzy systems to construct fuzzy rules to assist experts in detecting features and relations regarding the presence of autism in human beings. The model proposed in this paper works with a database generated through mobile devices that deals with diagnoses of autistic characteristics in human beings who answer a series of questions in a mobile application. The Bayesian model works with the construction of Gaussian fuzzy neurons in the first and logical neurons in the second layer of the model to form a fuzzy inference system connected to an artificial neural network that activates a robust output neuron. The new fuzzy neural network model was compared with traditional state-of-the-art machine learning models based on high-dimensional based on real-world data sets comprising the autism occurrence in children, adults, and adolescents. The results (97.73- Children/94.32-Adolescent/97.28-Adult) demonstrate the efficiency of our new method in determining children, adolescents, and adults with autistic traits (being among the top performers among all ML models tested), can generate knowledge about the dataset through fuzzy rules.

## Introduction

Autistic spectrum disorder (ASD) is characterized as a behavioral syndrome that impairs cognitive and relational aspects throughout the lives of children, adolescents, and adults. Striking features of autism are connected to irregular developmental patterns, autistic fascination with mechanical objects, repetitive responses to stimuli received by the environment, and resistance to any change in the life context. For example, extraordinary hearing sensitivity is common and causes them to be bothered by sounds that would not disturb a non-autistic person. ASD can be diagnosed as autistic disorder, childhood disintegrative disorder, generalized developmental disorder not specified (PDD-NOS), and Asperger syndrome. The symptoms of autism are confounded with the characteristics presented generally by the diagnosed patients with ASD, such as difficulty to interact socially, difficulty in communication, opting for repetitive use of language, and behavioral changes (Geschwind and Levitt 2007).

Early diagnosis of autism, especially in children, can help in approach to facilitate their living in society. For adolescents and adults, treatments and measures can be adapted to make everyday work or study life more accessible. However, this diagnosis may be complicated due to the features in common with other existing diseases. Thus, it becomes feasible to find models that assist in the diagnosis of autism (Teitelbaum et al. 1998).

Comprehensive studies are taking place in science to uncover factors that can cause autism, besides as ways to identify it early. An autistic child diagnosed in his early stages of life may suffer less from adjustments to the environment, just as his parents and those in their social life may be prepared to perform with peculiar features of this disease that impairs neural evolution in humans. Studies on this condition are expanded as society undergoes transformations and needs to identify more accurately and quickly features that may facilitate autistic diagnosis so that the impacts on this person’s life can be lessened (Jaliaawala and Khan 2019). Recent studies on autism range from patient data collection to computerized techniques and resources to assist in preliminary anomaly diagnoses. The use of techniques such as artificial intelligence, logical inference, intelligent estimators, and behavioral analysis (Rump et al. 2009) and practices contribute to a closer response to the real, helping doctors, parents, and even patients to obtain the best treatments (Mazurek et al. 2019).

Situations in the identification of autism are patterned and implemented in different medical offices. However, for ordinary people, these behaviors can be confused with different anomalies or even everyday situations experienced through the life stage of children, puberty, or adulthood. To help people identify traces of autism, research proposed by Thabtah (2018) was conducted to collect data related to human behaviors and situations that are evaluated at medical appointments to predict people with autism. These studies led to the creation of a free mobile application for this diagnosis to be presented preliminarily. The data collected in this application generated several studies and applications of artificial intelligence to improve autism prediction. Thabtah’s (2017a) abstract lists the central studies that seek to solve autism identification efficiently. The app that can be downloaded via mobile devices and includes topics that improve to distinguish the features of autism, converting a traditional form of data collection to create datasets for preparing the algorithms for detection of this kind of analysis. The application allows parents to see if the traces of this mental illness are in their adolescent (Thabtah 2017a). The mobile system works through a preliminary diagnosis based on renowned physicians’ experience in identifying autistic traits. The system provides a response that has a scale from 1 to 10 that is obtained by combining the responses provided in the application (Thabtah 2017a). The algorithms used in the first version of the app obtained consistent results in identifying autism in children, adults, and adolescents, but these models have reliable results and poor interpretability. Therefore, other intelligent techniques can aid in the accuracy of results, and the combination of intelligent techniques can allow more accurate results and more understandable interpretability for non-science people. To address the synergistic use of AI techniques, this paper proposes using a hybrid framework that has the interpretability of fuzzy rule-based systems and the learning provided by artificial neural networks (Pedrycz 1991). Fuzzy neural networks are layer-based models that can extract knowledge of a problem and update internal parameters of its structure through modern learning techniques to obtain solutions related to health treatments, such as immunotherapeutic treatments (Guimarães et al. 2018, 2019), detection of human falls (Souza et al. 2020), Parkinson (Guimarães et al. 2020), and the routine of companies and people (Pedrycz 2006).

In this algorithm, we highlight the regularization method based on re-sampling to select the characteristics most relevant to the model. Therefore, the proposed approach resembles the intelligent system schema proposed in Thabtah (2017a), allowing faster training and network formation because it is based on extreme learning machines (Huang et al. 2006) techniques.

This paper differs from our approach in de Campos Souza et al. (2019) because of the use of the fuzzification method in its Bayesian version to replace a data partition method that forms data clouds in the construction of the first-layer Gaussian neurons. The data density-based approach has been replaced by an incremental probabilistic approach, allowing the process of fuzzification of the model to be gradual and with probabilistic assessments of the problem data’s behavior. Therefore, this work adds the model’s ability to operate with the impact of the likelihood present in the fuzzification process dataset and, consequently, in the network architecture. The fuzzy models’ fuzzification process manipulates data that can delimit how hybrid models can interpret their results and make them closer to real-world situations. The fuzzification method alters the structure of an intelligent hybrid model, allowing the technique to produce associated impacts on the construction of elements (such as neurons) that make up the architecture of these models. Thus, it is expected that the choice of the data processing in this paper will directly affect the quality of the intelligent model outputs. Another factor that distinguishes it is related to the use of fuzzy neurons in the second layer of the model and the training method based on least squares employing enhanced regularization techniques based on Lasso technique. The differences presented between the previous model and the one proposed in this article focus on a probabilistic fuzzification approach, different from the approach that works with data density. The fuzzy rules generated in the second layer are selected according to a resource selection technique, allowing the model proposed in this article to identify the formation of the most relevant rules and use them for the model’s final responses. Using a probabilistic fuzzification approach, some neurons constructed with these methods may be redundant or unnecessary to assess the model. In this case, quality assurance is sought in the fuzzy rules generated by selecting the most significant fuzzy rules according to statistical criteria so that they are assertive in identifying the early diagnosis of autism.

This study’s purpose is to identify the possibility of autism in children, adolescents, and adults with a dataset formed by an application that allows the insertion of data to facilitate a person with traces of autism. This identification will be made through a fuzzy neural network model that will generate rules with a high degree of interpretability. These rules can facilitate disseminating knowledge about the problem, mainly by transforming data into interpretable information. Therefore, this study’s main objective is to evaluate data on patients who may or may not have autism and extract knowledge with a high degree of assertiveness to assist implementations of computerized systems that can be distributed to society. This paper’s main contribution to science is the construction of an intelligent model capable of identifying autism in adults, children, and adolescents. In addition to precise classification, it is expected to obtain knowledge of the dataset through fuzzy rules that represent transparent and readable relationships of the features for autism diagnosis as embedded in the problem assessment.

The paper is organized as follows: In Sect. 2 are the present terms of the literature that make up the study carried out in this paper. In Sect. 3, the fuzzy neural network model proposed to improve the accuracy and interpretability of autism identification is presented. In Sect. 4, the database used by the mobile device, configuration, and test results are shown. Finally, in Sect. 5, the conclusion about the experiments is presented to the reader of the paper.

## Literature review

### Autistic spectrum disorder

**Fig. 1:**
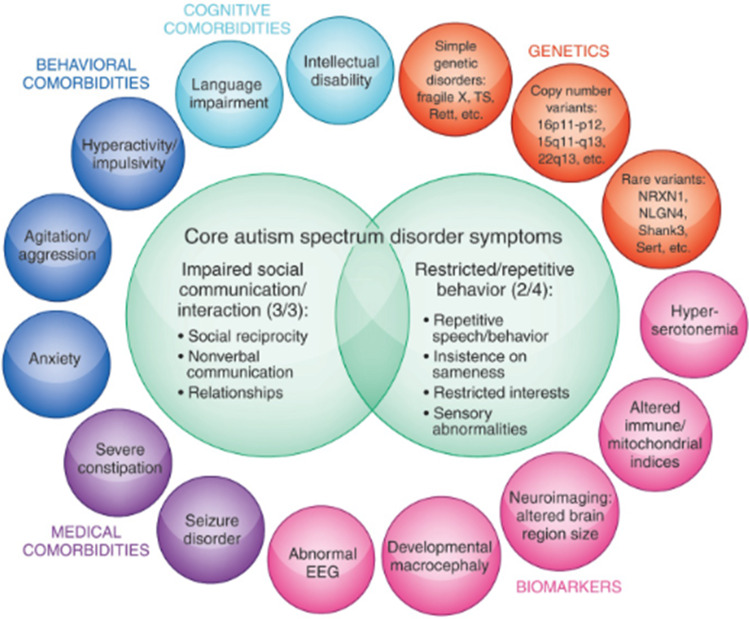
Autism symptoms. *Source*: Hewitson (2013)

Autism, a common form of autism spectrum disorder (ASD), is characterized by problems in communication, socialization, and behavior, usually diagnosed between 2 and 3 years of age in children (Thabtah 2017b). When the preliminary determination gives strong indications of autism in children or adolescents, the following impacts on the life of this youngster can be minimized with adequate treatment; however, it has always been difficult to obtain accurate data to diagnose health professionals the patient’s efficient way (Thabtah 2017a, b, 2018; Pedrycz 1991; Guimarães et al. 2018, 2019, 2020; Souza et al. 2020; Pedrycz 2006; de Campos Souza et al. 2019; Wall et al. 2012). In recent times, research has been improved, and with the aid of computational resources, it is possible to collect relevant data for new inferences about the study of ASD in adolescents. In Fig. 1, some symptoms are shown that adults can perceive in children or adolescents and also some behavioral information, disorders, and characteristics associated with the disease.

Although it is called childhood autism, it is essential to be aware that the disorder is a permanent condition, and it is named after the fact that the diagnosis of autism occurs typically in childhood. Therefore, an autistic child will become an adult with autism, but this aspect does not determine the individual’s living conditions. Early identification of the diagnosis can help parents understand the main limitations of this behavior and allow them to have fewer impacts during their life trajectory (Capps et al. 1998).

For Baron-Cohen et al. (2006) in adolescence, the symptoms vary according to the types of autism, and the person may have the difficulty of autonomy. That allows many parents to be unable to identify differences between children who are not communicative or who have an upward spectrum. At this stage, they can be affected by anxiety and depression, changing their habits of living with other people to live with only a restricted circle of people, or even to choose solitary activities without social interactions.

Finally, an adult who has autism also needs special care, mainly due to their difficulty adapting to some activities within the company’s work routine. His profile requires that the nature of the work be more individual to avoid more significant constraints on the adult (Billstedt et al. 2007).

The treatment for autism depends on which stage it was diagnosed in the person. In the early years, a speech therapist’s approaches can assist the development of nonverbal language, focused on facial expressions related to their behavior. As the person grows, older follow-up can be accomplished through techniques that can stimulate verbal communication. At this stage, the presence of professionals in the area of occupational therapy, psychologists, and social service professionals can accompany the society surrounding the autistic, preparing them to receive it without significant impacts. Therefore, the diagnosis’ detection becomes fundamental for the possibility of an adequate routine for the coexistence of the autistic in society (Frith 2003).

### Related works

The papers in the literature that address reasonable resolutions to aid in the diagnosis of autism. The application of models capable of identifying autistic problems and characteristics in a more abbreviated time allows people to be assisted and the impacts on autistic life to be minimized. In the research of Thabtah (2017b), a collection of algorithms implemented by the WEKA software (Hall et al. 2009) was used to evaluate the database of the first of four modules of the Generic Autism Diagnostic Observation Program. In their experiments, 16 data classification algorithms were used to classify people autistic. The models obtained high accuracy (extremely close to 100%), allowing the approach used by neural networks is a viable way of predicting people with autism. Other studies like Duda et al. (2014) have estimated autism in people of different points. The results obtained furthermore are relevant. However, the most suitable treatment by this work has drawn the attention of researchers such as Bone et al. (2015) which, in addition to using the techniques of neural networks, newborn leukocyte epigenomic markers were the object of research in Bahado-Singh et al. (2019) and Thabtah et al. (2019) that uses in the detection of autism regression techniques. In the work of Bertoncelli et al. (2019), the tests were performed in collections of adolescents with cerebral palsy. In De Campos Souza and Guimarães (2018), the use of fuzzy neural networks assisted in the prediction and comprehension of autistic children. Finally, articles presented in Carpenter et al. (2016) and Kamp-Becker et al. (2017) seek to deal with the reduction of time and complexity in obtaining more precise answers to the patients’ diagnosis.

Recent works have been promoted for the use of different intelligent models in aid of autism. The highlights go to the models of Thabtah et al. (2019) that used information gain and Chi-square testing techniques to determine the influences of a database collected in a mobile program for the creation of a classifier based on logistic regression analysis. In Thabtah and Peebles (2019), a machine learning model was created for the induction of rules for autism detection and, finally, the approach of Satu et al. (2019) applied intelligent techniques for the prediction of autism in a specific case in Bangladesh.

Also noteworthy are studies related to the use of fuzzy neural networks for the detection of autism in adolescents (de Campos Souza et al. 2019) and in adults (Guimarães et al. 2019). Also, in the work of de Campos Souza and Guimarães (2018), the main focus was on the children’s autism. The work proposed in this paper contrasts from the other papers already performed with fuzzy neural networks due to the characteristics related to the fuzzification process, which happens through a technique based on data grouping through a Bayesian (probabilistic-type) clustering approach as well as the type of activation functions in the third layer of the model. An enhanced regularization is embedded in the least-squares problem for learning the output weights connected by the neural network neuron: it tries to shrink as many as features toward 0, such that a kind of output weight and thus structural model reduction can be achieved. This, in turn, may decrease the likelihood of over-fitting for new cases for which autism should be identified.

### Artificial neural networks and fuzzy systems

Different applications logically require complex ANN characteristics, and in many circumstances, these characteristics are mainly speed and correctness. The use of ANNs is powerful because of its flexibility in application development. The responsibility of pattern recognition can be applied to several areas of knowledge by serving a general purpose, which humans are accustomed to, such as recognizing sounds, images, and smells. Artificial neural networks are complex mathematical models that are motivated by intelligent organisms’ neural structure and that extract knowledge through learning from data.

Fuzzy systems are based on fuzzy logic, proposed by Zadeh (1965). This work was motivated because of the wide variety of vague and uncertain information in making human decisions. The term fuzzy refers to things that are not clear or are vague. In the real world, we often encounter a situation where we cannot determine whether the state is true or false; its fuzzy logic provides precious flexibility for reasoning. In this way, we can consider the inaccuracies and uncertainties of any situation (Dubois 1980). A fuzzy system (FS) uses the concepts of fuzzy sets to express uncertainty and vagueness. The fuzzy sets are the basis for representing human knowledge in the form of linguistic terms such as LOW, OLD or TALL, which are not necessarily ‘modelable’ by single discrete numbers (e.g., a tall person may range from 180cm to 200cm, with starting a bit tall at around 175 cm—the possibility can well represent such a level of certainty degree expressed through fuzzy sets (Klir and Yuan 1995)). To establish fuzzy rules in a fuzzy system, interactions of input variables and intermodal relationships between the fuzzy sets can be modeled using different ways, such as t-norms, aggregation operators, and form analysis of association operators. Association functions are mathematical ways of representing values for the inference mechanism. Typically, a fuzzy system contains the following modalities (Kruse et al. 1994):

*Rule base:* It contains the set of rules and IF-THEN conditions provided by the experts in the problem or extracted from a database to govern the decision-making system. The rules are representable as linguistically readable information.

*Fuzzification:* It is used to convert entries, that is, sharp numbers into certainty degrees based on fuzzy set activation levels. The precise inputs are the same inputs present in a database, such as a temperature, pressure, rpm, etc. A fuzzy set typically is a unimodal functional mapping from the input space to a membership value in [0, 1].

*Inference Engine:* Determines the degree of matching of the current fuzzified input for each rule and decides which rules are to be triggered according to the input field. The fired rules are combined to form the control actions, which are parts of the rule consequents.

*Defuzzification:* It is used to convert the consequent activations (can be again fuzzy sets or numerical functions) obtained by the inference mechanism to a crisp value. There are several defuzzification methods available, and the most suitable one is used with a specific expert system to reduce the error (Pedrycz 1991).

*Membership function: *A graph that defines how each point in the input space is mapped to a membership value between 0 and 1.

#### Fuzzy neural networks

Fuzzy neural networks are described by neural networks constructed of fuzzy neurons (Pedrycz and Gomide 2007). A fuzzy neural network or neuro-fuzzy system is a learning mechanism that determines the parameters of a fuzzy system (i.e., fuzzy sets, fuzzy rules) by investigating methods of artificial neural network approach and training. Its main functionality is to perform the extraction of knowledge from a database through fuzzy rule sets that determine rules of logical inference able to describe the field of the analyzed problem. Neural networks can only be used in solving a complex context if a sufficient number of practical examples express the problem. These observations are used to train the model. On the one hand, no prior knowledge about the problem needs to be given. On the other hand, however, it is not easy to extract comprehensible rules from the neural network structure. The fuzzy systems come to remedy some of these weaknesses, and therefore, the union of the two techniques creates powerful models for solving complex problems (Pedrycz and Gomide 2007).

Compared to a typical neural network, the connection weights and propagation functions, and activation of diffuse neural networks differ significantly. The frequent use of fuzzy systems and neural network training techniques can lead to more extended training to solve big data problems. A neuro-fuzzy system based on an underlying fuzzy system is trained through a data-based learning method derived from the neural network theory. The fuzzy neural network uses the problem data to define its architecture, allowing fuzzification techniques to find efficient parameters to model the intelligent technique’s structure. Each layer of the hybrid models can act with different functions, such as fuzzification, defuzzification, rule aggregation, model training, feature extraction, among others (Pedrycz and Gomide 2007).

These intelligent models can act in solving various problems due to the freedom of their actions and the ease of use of their techniques (de Campos Souza 2020). They are prominent in robotic problem solving as in the Chatterjee et al. model. (Chatterjee et al. 2005) to assist in robot control of a robot, or even to identify fraudulent aspects in financial reports as in the model of Lin et al. (2003). However, it should be noted that these models have a different role in the resolution of problems related to health, such as Allen et al. (2015) model that works in the health monitoring system, specifically in the variable air volume or the model of Pavlopoulos et al. (2000) that uses the hybrid model based on texture analysis of ultrasonic images. Other recent works highlighting pulsar detection (de Campos Souza et al. 2019) and the recent work by De Campos Souza et al., which deals with the pattern classification data (de Campos Souza et al. 2019).

Also noteworthy in the health area is the performance of fuzzy neural networks in coronary disease problems (Akay et al. 1994), Parkinson (Lee and Lim 2012), breast cancer (Silva Araújo et al. 2019), and lymphoma (Ando et al. 2002). Therefore, there is a great use of the model to act in precise diagnoses in the health area.

## Fuzzy neural network for autism spectrum disorder test-BFNNRELU

This section will present the main factors present in the proposed model to solve problems related to the database related to autism, highlighting the architecture of the model, its parameters, and training methods.

### Network architecture

The fuzzy neural network described in this paper is composed of three layers. In the first layer, fuzzification is used through the concept of Bayesian probability. The centers of the clusters are used to create fuzzy Gaussian neurons in the first layer. The $\sigma$ value complements the construction of the neuron, and it is obtained through the fuzzification technique of the model. The weights and biases of the first layer’s neurons are randomly defined in the range of 0 to 1.

In the second layer, the logical neurons of the andneuron type are applied (Eq. 19). These neurons have weights, and activation functions determined at random and fuzzy operators are used to aggregate the first layer of the model’s elements. Andneuron (Pedrycz 1991) is used to construct fuzzy neural networks in the second layer to solve pattern recognition problems and bring interpretability to the model.

**Fig. 2:**
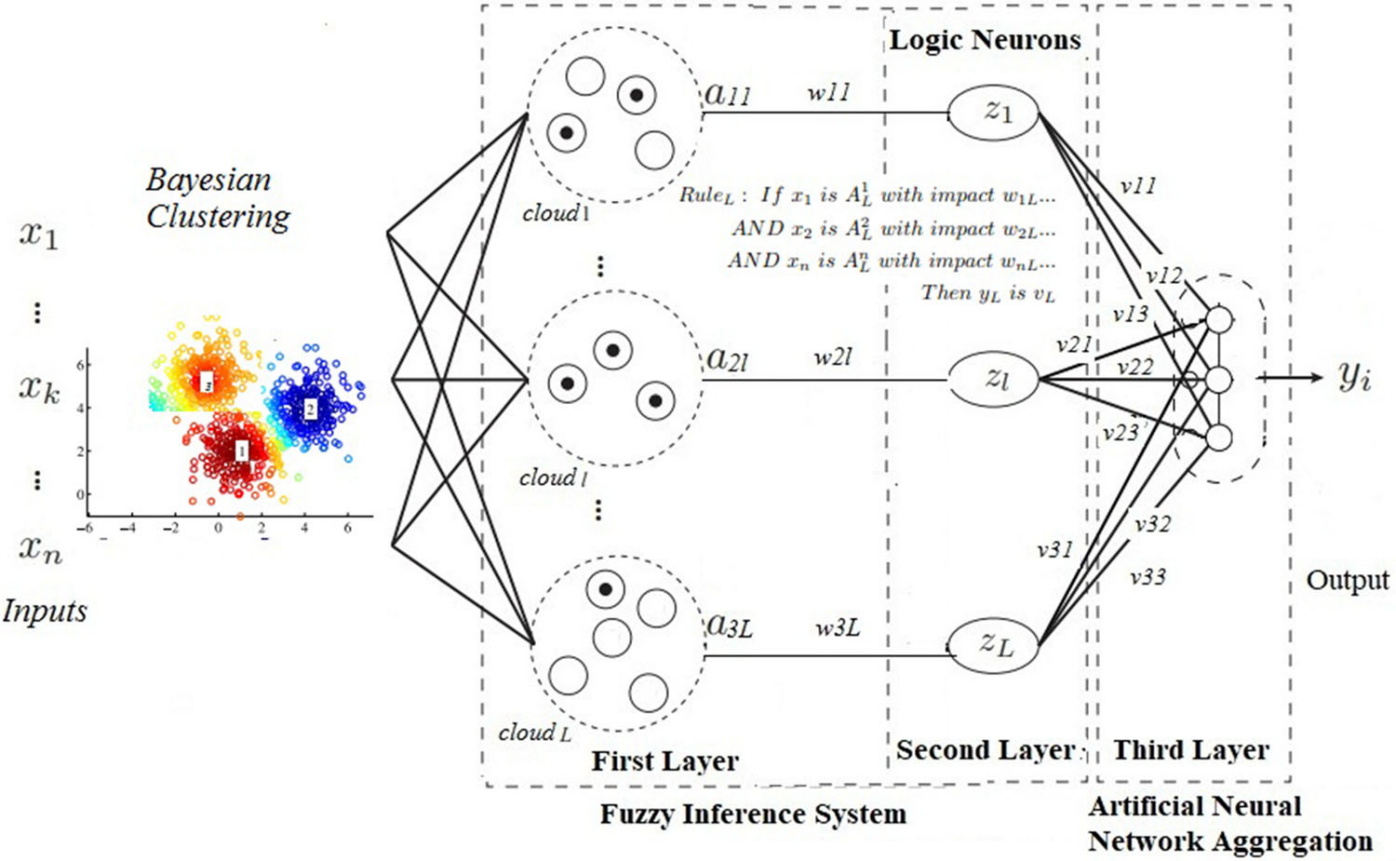
FNN architecture

To define the weights that connect the second layer with the output layer, the concept of extreme learning machine proposed by Huang et al. (2006) is used to act on the neuron with a rectifier nonlinearities activation function (LeakyReLU) (Maas et al. 2013). Figure 2 illustrates the feedforward topology of the fuzzy neural networks considered in this paper. In Fig. 2, it is possible to highlight the centers (**c**) and sigmas ($\sigma$) originating from the fuzzification technique and the weights of the fuzzy neurons (**w**).

#### First layer—input data and fuzzification through Bayesian clustering

The first layer is composed of neurons whose activation functions are fuzzy set association functions defined for the input variables. This fuzzification approach is based on the essence of the problem data and its probability of forming the decision grid and transforming the problem values into fuzzy representations. For each input variable $x_{ij}$, *L* clouds are defined $A_{lj}$, *l* = 1 ...*L* whose membership functions are the activation functions of the corresponding neurons. The clouds are thereby formed through the extraction of clusters in the high-dimensional space, for which we apply a Bayesian approach in order to obtain an appropriate granularity of the input feature space in a probabilistic manner.

The problem of clustering using fuzzy rules has great representativeness in the concepts of fuzzy c-means (Bezdek 2013), which can be expressed by the minimization of:

1 $$\begin{aligned} J({\mathbf {X}},{\mathbf {U}},{\mathbf {Y}}) = \sum _{n=1}^N \sum _{c=1}^C u_{nc}^m \, d({\mathbf {x}}_n,{\mathbf {y}}_c)^2 \end{aligned}$$

where $x_n$ is the data point (with *N* data points in sum), $y_c$ is the prototype of each cluster (with *C* clusters in sum), $\left\{ u_{nc}\right\}$ is the membership degree of point *n* to cluster *c*, *m* is a fuzzifier parameter (typically set to 2), and $d({\mathbf {x}}_n,{\mathbf {y}}_c) = \Vert {\mathbf {x}}_n - {\mathbf {y}}_c \Vert$ is a distance function, typically the Euclidean distance is used (Glenn et al. 2014).

To complement the knowledge about fuzzy grouping, it is necessary to present the values of memberships and cluster prototypes through differentiation of the objective function with Lagrange multiplier constraints. These elements can be expressed by Glenn et al. (2014):

2 $$\begin{aligned} u_{nc}= & {} \frac{ (1/d({\mathbf {x}}_n,{\mathbf {y}}_c)^2)^{1/(m-1)} }{ \sum _{k=1}^C \left( 1/d(\mathbf {x}_n,{\mathbf {y}}_k)^2 \right) ^{1/(m-1)} } \end{aligned}$$

3 $$\begin{aligned} {\mathbf {y}}_c= & {} \frac{1}{\sum _{n=1}^N u_{nc}^m }\sum _{n=1}^N u_{nc}^m {\mathbf {x}}_n. \end{aligned}$$

Concerning the infinite Bayesian fuzzy clustering (IBFC) strategy employed in this paper, it is expected that the association of unknown sets $u_ {nc}$ and definition of expected prototypes as random variables. Given the specific limitations and uncertainties associated with each random variable in real complex problems, we selected an appropriate probability distribution for each, following guidelines established by Bayesian theory. We then infer the most likely value of these variables, given the observed data. IBFC can be extended to represent the number of clusters *C* as a stochastic variable, making it a viable strategy for becoming nonparametric (Glenn et al. 2014).

The initial concept, called Bayesian fuzzy clustering (BFC), uses a data probability distribution called fuzzy data likelihood (FDL) and can be described as (Glenn et al. 2014):

4 $$\begin{aligned}&p({\mathbf {X}}|{\mathbf {U}},{\mathbf {Y}}) = \prod _{n=1}^N \mathrm{FDL}({\mathbf {x}}_n|{\mathbf {u}}_n,{\mathbf {Y}}) \nonumber \\&\quad = \prod _{n=1}^N \frac{1}{Z({\mathbf {u}}_n,m,{\mathbf {Y}})} \prod _{c=1}^C {\mathcal {N}}({\mathbf {x}}_n|{\varvec{\mu }}= {\mathbf {y}}_c, \varvec{\Lambda }= u_{nc}^m\, {\mathbf {I}}) \end{aligned}$$

where $y_c$ is the set prototypes and $u_n$ is the set memberships, which are two groups of unknown variables in fuzzy clustering problem. The technique also uses the concept of fuzzy cluster prior (FCP) that is the prior distribution for the cluster membership defined as (Glenn et al. 2014):

5 $$\begin{aligned}&{\tilde{p}}({\mathbf {U}}|{\mathbf {Y}}) = \prod _{n=1}^N \mathrm{FCP}({\mathbf {u}}_n|{\mathbf {Y}}) \nonumber \\&\quad =\prod _{n=1}^N Z({\mathbf {u}}_n,m,{\mathbf {Y}}) \left( \prod _{c=1}^C u_{nc}^{-mD/2} \right) \mathrm{Dirichlet}({\mathbf {u}}_n|\varvec{\alpha }) \end{aligned}$$

moreover, finally the technique employs the concepts of Gaussian prior distribution on cluster prototypes (Glenn et al. 2014):

6 $$\begin{aligned} p({\mathbf {Y}}) = \prod _{c=1}^C {\mathcal {N}}(\mathbf {y}_c|{\varvec{\mu }}_y,\mathbf {\Sigma }_y) \end{aligned}$$

For the interpretation of Eqs. (4), (5) and (6) consider (Glenn et al. 2014): *N* = number of data points, *C* = number of clusters, *D* is the dimensionality of the data, $u_{nc}$ is the membership of data point $x_n$ in cluster *c*, *m* is the fuzzifier, $y_c$ are the cluster prototypes. The arguments to the probability density functions are grouped into the *DxN* matrix of data points **X**=[$x_1$...$x_N$], memberships **U** with dimensions *CxN* and prototypes **Y** = [$y_1$...$y_C$] with dimensions *DxC* (Glenn et al. 2014). **I** is the *D*-dimensional identity matrix, **Z** ($\mathbf{u }_n$, *m*, **Y**) is a normalization constant obtained through (product of Gaussian likelihood functions):

7 $$\begin{aligned}&Z({\mathbf {u}}_n,m,{\mathbf {Y}}) = (2\pi )^{-\frac{D}{2}(C-1)} \left( \prod _{c=1}^C u_{nc}^m \right) ^\frac{D}{2} \left( \sum _{c=1}^C u_{nc}^m\right) ^{-\frac{D}{2}} \nonumber \\&\quad \times \exp \left\{ -\frac{1}{2} \left( \sum _{c=1}^C u_{nc}^m {\mathbf {y}}_c^T {\mathbf {y}}_c - \frac{ ||\sum _{c=1}^C u_{nc}^m {\mathbf {y}}_c ||^2 }{ \sum _{c=1}^C u_{nc}^m } \right) \right\} \end{aligned}$$

where $\lambda =u_{nc}^m$ is the precision of each of the normal component specific to each data point, ${\varvec{\mu }}= \mathbf {y}_c$ are the cluster prototypes, see also Glenn et al. (2014) for further details.

In the FDL, the data have a probability equivalent to the product of the standard number’s clusters probabilities, each with distinct precision. The accuracy of each of the conventional components present in the cluster is distinguished for each model input sample. That allows assigning a low association value, therefore producing a high variance in the regular components away from the probability. Consequently, each data point can be considered as possessing its generating probability distribution. However, the standard probabilities are classified so that they share average values that are the prototypes of the clusters (Glenn et al. 2014).

It is assumed that the relationship variables have an early maturing distribution. We call it prior, the FCP. This former consists of three factors that are determined by $F_1$ = ($Z(\mathbf {u}_n,m,{\mathbf {Y}})$), $F_2$ = $\left( \prod _{c=1}^C u_{nc}^{-mD/2} \right)$, and $F_3$ = ($\mathrm{Dirichlet}(\mathbf {u}_n|\varvec{\alpha })$).

The first two factors are a counterbalance to the likelihood of $F_1$ data, ignoring exactly the normalization constant of the FDL. Also, the $F_2$ factor occurs because the approach’s Gaussian components will support high correlation values. The individual factors of $F_2$, however, are more significant for small connection values that end up being canceled, making the joint distribution more agnostic concerning the association values. Because of the negative value of the exponent in $F_2$, however, the FCP cannot be normalized over the interval [0,1]. The FCP within this context can be seen as a mathematical convenience to accurately replicate the behavior of the FCM by the Bayesian model. Subsequently, the $F_3$ factor is called the Dirichlet likelihood and is parameterized by the vector $\alpha$, defined as (Glenn et al. 2014):

8 $$\begin{aligned} \mathrm{Dirichlet}({\mathbf {x}}|\varvec{\alpha }) = \frac{\Gamma (\sum _{k=1}^K \alpha _k)}{\prod _{k=1}^K \Gamma (\alpha _k)} \prod _{k=1}^K x_k^{\alpha _k-1} \end{aligned}$$

where $\alpha = mD/2-1$ and the domain of the standard simplex, $x_k \ge 0$ for all $k=1,\ldots , K \sum _{k=1}^K x_k = 1$. The addition of the Dirichlet factor satisfactorily expresses the positivity and sum-of-one restrictions in the associations. Also, it provides additional flexibility and capacity to the clustering algorithm, while including FCM as a sub-example of the usual method.

Finally, the BFC model assigns Gaussian before each of the cluster prototype parameters. The hyperparameters of this arrangement can be set in the experimental Bayes procedure to handle the average of the dataset. They can be defined as:

9 $$\begin{aligned} {\varvec{\mu }}_y = \frac{1}{N} \sum _{n=1}^N {\mathbf {x}}_n \end{aligned}$$

also, an extensive covariance:

10 $$\begin{aligned} \Sigma _y = \frac{\gamma }{N} \sum _{n=1}^N ({\mathbf {x}}_n - {\varvec{\mu }}_y) ({\mathbf {x}}_n - {\varvec{\mu }}_y)^T \end{aligned}$$

where $\gamma$ in this paper was handled assuming the value of 3, such as in Gleen et al. (2014).

When considering the probability of log-likelihood the complete BFC model as an objective function of a fuzzy cluster, a regularization term is obtained through the cluster prototype. A different factor to consider in the fuzzification technique is the joint likelihood of the data and parameters. It can be obtained by multiplying fuzzy data likelihood, fuzzy cluster prior to the Gaussian prior distribution on cluster prototypes reported earlier in the paper. Thus, it is expressed by Glenn et al. (2014):

11 $$\begin{aligned}&p({\mathbf {X}},{\mathbf {U}},{\mathbf {Y}}) = p({\mathbf {X}}|\mathbf {U},{\mathbf {Y}}) {\tilde{p}}({\mathbf {U}}|\mathbf {Y}) p({\mathbf {Y}}) \nonumber \\&\quad \propto \exp \left\{ -\frac{1}{2} \sum _{n=1}^N \sum _{c=1}^C u_{nc}^m \Vert {\mathbf {x}}_n - \mathbf {y}_c \Vert ^2 \right\} \left[ \prod _{n=1}^N \prod _{c=1}^C u_{nc}^{\alpha _c-1}\right. \nonumber \\&\quad \left. \times \exp \left\{ -\frac{1}{2} \sum _{c=1}^C ({\mathbf {y}}_c - \mu _y)^T \mathbf {\Sigma }_y^{-1} ({\mathbf {y}}_c - \mu _y) \right\} \right] \end{aligned}$$

which is proportional to the posterior distribution of the parameters, $p({\mathbf {U}},{\mathbf {Y}}|{\mathbf {X}}) \propto p({\mathbf {X}},{\mathbf {U}},{\mathbf {Y}})$. The objective function form of the joint likelihood is the negative of its logarithm and thus can be expressed by:

12 $$\begin{aligned} J({\mathbf {X}},{\mathbf {U}},{\mathbf {Y}})= & {} \sum _{n=1}^N \sum _{c=1}^C u_{nc}^m \Vert {\mathbf {x}}_n - \mathbf {y}_c \Vert ^2 \nonumber \\&\quad -\; 2 \sum _{n=1}^N \sum _{c=1}^C (\alpha _c-1)\log (u_{nc}) \nonumber \\&\quad +\sum _{c=1}^C ({\mathbf {y}}_c - \mu _y)^T \mathbf {\Sigma }_y^{-1} ({\mathbf {y}}_c - \mu _y) \end{aligned}$$

When the clustering approach averages the objective value over the number of model components, it can remove some dependency on the model size. Subsequently, it is possible to use a size penalty to discriminate larger models that are otherwise equally good. By redefining the objective function of the Bayesian cluster technique, we can rewrite (12) by including a penalty:

13 $$\begin{aligned}&J({\mathbf {X}},{\mathbf {U}},{\mathbf {Y}},{\mathbf {C}}) = \frac{1}{C} \sum _{n=1}^N \sum _{c=1}^C u_{nc}^m \Vert {\mathbf {x}}_n - {\mathbf {y}}_c \Vert ^2 \nonumber \\&\quad -\; \frac{2}{C} \sum _{n=1}^N \sum _{c=1}^C (\alpha _c-1)\log (u_{nc}) \nonumber \\&\quad +\; \frac{1}{C} \sum _{c=1}^C ({\mathbf {y}}_c - {\varvec{\mu }}_y)^T \mathbf {\Sigma }_y^{-1} ({\mathbf {y}}_c - {\varvec{\mu }}_y) + \mathrm{Penalty}(C). \quad \end{aligned}$$

If the fuzzification technique considers the basic form of this objective as the prototype for the negative log-likelihood, the model called infinite Bayesian fuzzy clustering (IBFC) is obtained. It consists of a data likelihood and can be expressed as (Glenn et al. 2014):

14 $$\begin{aligned} p({\mathbf {X}}|{\mathbf {U}},{\mathbf {Y}},C) = \prod _{n=1}^N \frac{1}{Z_{\mathbf {X}}} \prod _{c=1}^C {\mathcal {N}}\left( {\mathbf {x}}_n|{\varvec{\mu }}= {\mathbf {y}}_c, \varvec{\Lambda }= \frac{1}{C}u_{nc}^m\, I \right) \nonumber \\ \end{aligned}$$

A prior distribution over membership values (Glenn et al. 2014):

15 $$\begin{aligned} {\tilde{p}}({\mathbf {U}}|{\mathbf {Y}},C)= & {} \prod _{n=1}^N \left[ Z_{\mathbf {X}}\left( \prod _{n=1}^C \frac{u_{nc}^m}{C}^{\frac{D}{2}} \right) \mathrm{Dirichlet}({\mathbf {u}}_n|\varvec{\alpha })^{\frac{1}{C}} \right] \nonumber \\&\times \left( C^{-C\frac{D}{2}} \right) \exp \left\{ \frac{\beta N}{C}\right\} \end{aligned}$$

Gaussian prior on the cluster prototypes (Glenn et al. 2014):

16 $$\begin{aligned} p({\mathbf {Y}}|C) = \prod _{c=1}^C {\mathcal {N}}({\mathbf {y}}_c|{\varvec{\mu }}_y,C\mathbf {\Sigma }_y) \end{aligned}$$

Finally, Poisson prior on the number of clusters define the Bayesian approach using *C* as a random variable (Glenn et al. 2014):

17 $$\begin{aligned} P(C) = \mathrm{Poisson}\;(C|\Theta ) = \frac{\Theta ^C}{C!} \exp \left\{ -\Theta \right\} . \end{aligned}$$

The probability of logarithmic joining of the IBFC model formed from Eq. (14,15,16,17) product log can be:

18 $$\begin{aligned}&\log p({\mathbf {X}},{\mathbf {U}},{\mathbf {Y}},C) \propto -\frac{1}{2C} \sum _{n=1}^N \sum _{c=1}^C u_{nc}^m \Vert {\mathbf {x}}_n - {\mathbf {y}}_c \Vert ^2 \nonumber \\&\quad +\;\frac{1}{C} \sum _{n=1}^N \log \mathrm{Dirichlet}(\mathbf {u}_n|\varvec{\alpha }) + \frac{\beta N}{C} \nonumber \\&\quad -\;\frac{1}{2C} \sum _{c=1}^C ({\mathbf {y}}_c - {\varvec{\mu }}_y)^T {\varvec{\Sigma }}_y^{-1} (\mathbf {y}_c - {\varvec{\mu }}_y) \nonumber \\&\quad +\; C \log \lambda - \sum _{i=1}^C \log (i). \end{aligned}$$

It is taking the negative of Eq. 18 presents an objective function formulation that answers to the objective proposed in Eq. 13, where the earlier one in C and the dispersion terms of the model combine to become the proposed penalty function. Therefore, this paper will address the IBFC for the first layer of the model’s fuzzification process.

This Bayesian cluster approach was used in several concepts in the literature, such as student performance evaluation (Ramanathan et al. 2019), brain tumor classification, and segmentation (Raju et al. 2019), medical images (Yadav 2019), among others. It should be noted that this approach has already been used in hybrid models that use neural networks and fuzzy systems. However, unlike in this paper, only BFC was used to solve regression problems (Souza et al. 2019). Therefore, it is plausible to infer that the technique is capable of acting dynamically and precisely in cluster identification processes and downstream classification tasks, a relevant factor for the fuzzy neural network process proposed in this paper.

**Fig. 3:**
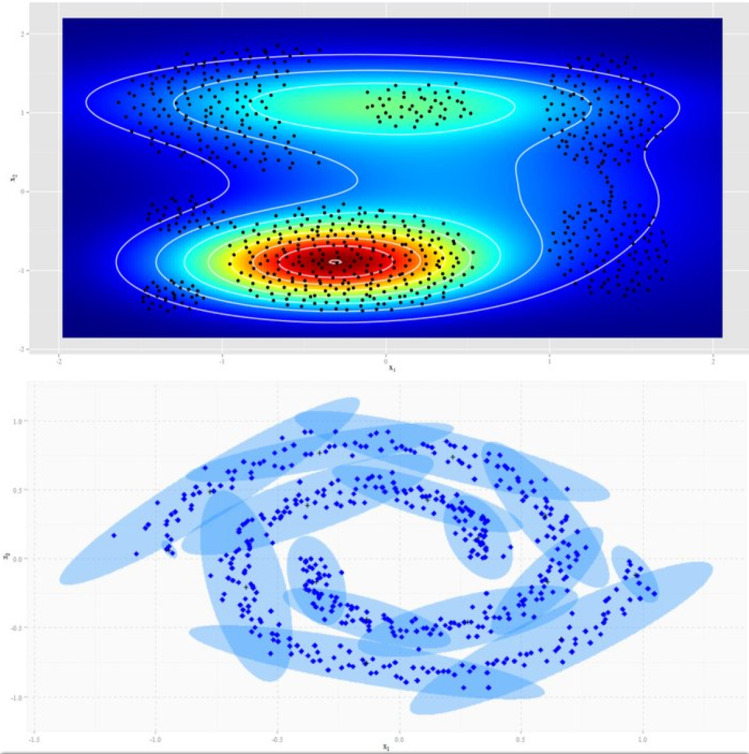
Example of the Bayesian cluster approach defining membership functions

Figure 3 presents an example of the approach proposed in synthetic clusters.

Thus, the outputs of the first layer are the membership degrees defined in an fuzzy probability way associated with the input values, i.e., $a_{jl}$ = $\varpi ^A_l$ for *j* = 1...*N* and *l* = 1 ...*L*, where *N* is the number of inputs and *L* is the number of fuzzy sets for each input results by IBFC (Eq. 16).

#### Second layer—fuzzy rules

The second layer is composed of *L* fuzzy AND-neurons. Each neuron performs a weighted aggregation of all of the first layer outputs using fuzzy operators called t-norm and s-norm. This aggregation is performed using the weights $w_{il}$ (for *i* = 1 ...*N* and *l* = 1 ...*L*) defined in the range of 0 to 1. For each input variable *j*, only one first layer output $a_{jl}$ is defined as input of the *l*-th neuron. So that **w** is sparse; each neuron of the second layer is associated with an input variable.

Fuzzy logic neurons can be used to replace artificial neurons in original structures. The logical neurons used in the second layer of the model are of the andneuron type, where the input signals are individually combined with the weights and performed the subsequent global aggregation. The andneuron used in this work can be expressed as (Pedrycz 1991):

19 $$\begin{aligned} z=\mathrm{AND} (w;a)=T^n_{i=1} (w_i \ s \ a_i) \end{aligned}$$

where *T* is a *t-norm* and *s* denotes an *s-norm*. In our case, in order to meet the probabilistic clusters extracted from the Bayesian approach, the t-norm is realized through the product and the s-norm through the probabilistic sum.

Fuzzy rules can be extracted from the AND-neuron according to the following example:

20 $$\begin{aligned} \begin{aligned} \mathrm{Rule}_1: \ \mathrm{If} \ x_{1} \ \mathrm{is} \ A_1^1 \ {\text {with impact}} \ w_{11}\ldots \\ \mathrm{AND} \ x_{2} \ \mathrm{is} \ A_1^2 \ {\text {with impact}} \ w_{21} \ldots \\ \mathrm{AND} \ x_{n} \ \mathrm{is} \ A_1^n \ {\text {with impact}} \ w_{n1} \ldots \\ \mathrm{Then} \ y_1 \ \mathrm{is} \ v_1\\ \mathrm{Rule}_L: \ \mathrm{If} \ x_{1} \ \mathrm{is} \ A_L^1 \ {\text {with impact}} \ w_{1L} \ldots \\ \mathrm{AND} \ x_{2} \ \mathrm{is} \ A_L^2 \ {\text {with impact}} \ w_{2L} \ldots \\ \mathrm{AND} \ x_{n} \ \mathrm{is} \ A_L^n \ {\text {with impact}} \ w_{nL} \ldots \\ \mathrm{Then} \ y_L \ \mathrm{is} \ v_L\\ \end{aligned} \end{aligned}$$

with $A_i^1,\ldots , A_i^n$ fuzzy sets represented as linguistic terms for the *n* inputs appearing in the *i*th rules, and $y_1,\ldots ,y_L$ are consequent terms, in our case classification responses (either 0 = no autism or 1 = autism); thus, each rule stands for one concrete readable relationship between the input features and the presence of autism; whenever a weight $w_{ij}$ is close to 0, the corresponding antecedent part can be neglected when showing the rules to experts; this reduces the rule length and increases the transparency and readability of the rules. Hence, these rules allow the creation of a building base for an expert system (Caminhas et al. 1999).

#### Third layer—artificial neural network

Finally, the output layer (third output) is composed of one neuron whose activation functions are leaky ReLU (Eq. 21) (Maas et al. 2013).

The activation functions allow small changes in weights and bias to cause only a slight change in output. Activation functions are a crucial element of artificial neural networks. They decide whether a neuron should be activated or not. That is, whether the information the neuron is receiving is relevant to the information provided or should be ignored.

The activation function is the nonlinear transformation along with the input signal. This transformed output is then sent to the next layer of neurons as input. When we do not have the activation function, the weights and bias do a linear transformation. A linear equation is simple to solve but is limited in its ability to solve complex problems (Leshno et al. 1993). They are indispensable to give a representative capability for fuzzy neural networks by proposing a nonlinearity component. On the other hand, with this power, some difficulties appear, mainly due to the diversified variety of activation functions that can change the effectiveness of their specific activities properties of the database to which the submitted model. In general, by introducing nonlinear activation, the cost outside of the neuron is no longer convex, making optimization more complicated (as iterations can become stuck in local optima). In problems that use parameterization by descent gradients, nonlinearity makes it more identifiable which elements need adjustment (Karlik and Olgac 2011).

The data and the nature of the information interfere with the classifier responses that have extreme learning machine-based training. In general, the activation functions must answer that if it must be nonlinear, if the activation function is linear, there must be a maximum and minimum output value of the activation function to limit the limit of weights and activation functions in the model and by the order, they should be continuous and smooth. Otherwise, it does not change the signal across the range of independent variables, and the model can avoid local minima (Liu et al. 2014).

We employ an improved version of the ReLU activation function (Nair and Hinton 2010) due to the insertion of a small linear component at the input of the neuron. This type of change allows small changes to be noticed, and neurons relevant to the model are not discarded. Its function is expressed by Maas et al. (2013):

21 $$\begin{aligned} f_\mathrm{LeakyReLU}(x,\kappa )= \max (\kappa x,x) \end{aligned}$$

where $\kappa$ is a fixed parameter in range (0.01, +$\infty$). In an original paper (proposed to address acoustics problems), the authors suggest setting $\kappa$ to a large number like 100. Nevertheless, in this paper, the nearest zero numbers will be adopted (0.01).^1^ Leaky ReLU function has two advantages over conventional ReLU function:

- It fixes the ReLU problem, as it does not have zero-slope components when *x* becomes negative.
- Besides that having the mean activation be adjacent to 0 makes training faster.

The output of the model is:

22 $$\begin{aligned} y= \Upsilon \sum _{j=0}^{l} f_\mathrm{LeakyReLU}(z_l, \ v_l) \end{aligned}$$

where $z_0$ = 1, $v_0$ is the bias, and $z_j$ and $v_j$, *j* = 1, ..., *l* are the output of each fuzzy neuron of the second layer and their corresponding weight, respectively. The $\Upsilon$ function can be represented by:

23 $$\begin{aligned} \Upsilon =\left\{ \begin{matrix}-1 &{}, \mathrm{if}\ \sum _{j=0}^{l} f_\mathrm{LeakyReLU}(z_l, \ v_l) < 0 \\ 1 &{}, \mathrm{if}\ \sum _{j=0}^{l} f_\mathrm{LeakyReLU}(z_l, \ v_l) > 0 \end{matrix}\right. , \end{aligned}$$

### Training of the fuzzy neural network

Membership functions in the first layer of the FNN are assumed as Gaussian, composed within the centers acquired by the method of granularization (IBFC) of the input space and by ($\sigma$) established in a value ranging from [0.1–1]. The number of neurons *L* in the first layer is determined according to the probability nature of input data, and through the numbers of partitions ($\rho$), explained parametrically through the Bayesian clustering approach. These actions are the central contrast of other models of fuzzy neural networks that accomplished in solving problems of autism because in their first layer procedures were used that had an exponential relation between the number of features of the problem and the number of membership functions determined in the initial steps. Therefore, because it is an exponential problem, the model’s training generated a very high computational cost, which may even lead to virtual memory overflow in the case of either high input dimensionality *n* or *L* being large (due to $n^L$ possible connections).

The fuzzification proposal used in this paper implements separations of the input space, following the logic definition of creating data nodes. The centers of these produced clouds make up the Gaussian activation functions of the fuzzy neurons. These changes will allow the adaptation of the data according to the dataset submitted to the model, allowing a more independent and data-centered approach. The second layer performs the aggregation of the *L* neurons from the first layer through the AND-neuron.

After the construction of the *L* AND-neurons, we try to eliminate as many neurons as possible according to actual learning problem at hand, that is, by taking into account also the target *y* (and not only the input features as done by Bayesian clustering a priorly) and to check which neurons are not crucial for explaining *y* (to achieve a good model accuracy). Therefore, we employ the usage of the Bolasso algorithm (Bach 2008), which is executed by applying LARS (Hastie et al. 2009) for obtaining the most significant neurons (called $L_s$). The final network architecture is thus defined through a feature extraction technique based on $L_1$ regularization and resampling. This approach performs a regularization of the training cost function by shrinking the coefficients of those neuron output weights to 0, which are unimportant for explaining *y*. In this sense, neurons with 0 weights can be discarded from the network.

In particular, the learning algorithm assumes that the output hidden layer composed of the candidate neurons can be written as (de Campos Souza et al. 2018):

24 $$\begin{aligned} f(x_i)=\sum _{i=o}^{L_p} v_iz_i(x_i)=z(x_i)v \end{aligned}$$

where **v** = [$v_0, v_1, v_2, \ldots , v_{L\rho }$] is the weight vector of the output layer and **z** ($x_i$) = [$z_0, z_1 (x_i), z_2 (x_i)\ldots z_{L\rho } (x_i$) ] the output vector of the second layer, for $z_0$ = 1. In this context, **z** ($x_i$) is considered as the nonlinear mapping of the input space for a space of fuzzy characteristics of dimension $L_\rho$ (de Campos Souza et al. 2018).

The solution of the least-squares problem on the linear consequent parameters $\mathbf {v}$ is achieved through the Moore–Penrose pseudo-inverse (de Campos Souza et al. 2018):

25 $$\begin{aligned} \mathbf {v} = \mathbf {Z}^{+}\mathbf {y} \end{aligned}$$

with $\mathbf {Z}^{+} = (\mathbf {Z}^T\mathbf {Z})^{-1}$. Therefore, the synaptic weights for the fuzzy rules and consequently for the neural network with a single neuron are defined in an agile and efficient way through a closed-loop formula, avoiding unnecessary optimization iterations in the fuzzy neural network structure.

The neurons (rules) selected by the combined LARS and bootstrap algorithm can be interpreted as being more meaningful to solve the autism problem, so from the latter, the synaptic weights of the artificial neural network present in the third layer of the model can be finally generated.

The LARS method (Efron et al. 2004) consists of first choosing between covariates, that is, between vectors $x_i$ (columns of matrix **X**) the one that has the most considerable correlation with data vector **y** (outputs). Two highly correlated covariates must be part of the model, or by the principle of parsimony, it is reasonable for only one of them to be present in the model. Therefore, the use of a decision factor is fundamental to choose sufficient criteria in deciding variables that should be contained in the model. Thus, algorithms must adopt some criteria to define the correlation level between two vectors analyzed to select values pertinent to the final model. LARS uses a process control parameter ($\lambda$), making no distinction between positively and negatively correlated. We conclude that the value of $\lambda$ must be small for the process to be able to detect highly correlated covariates. When judge a set of *K* distinct samples ($x_i, y_i$), where $x_i = [x_{i1}, x_{i2}\ldots x_{iN}$] $\in$ ${\mathbb {R}}$ and $y_i$ $\in$ ${\mathbb {R}}$ for *i* = 1...*K*, the cost function of this regression algorithm can be described as (de Campos Souza et al. 2018):

26 $$\begin{aligned} \sum _{i=1}^{K} \left\| \mathbf {z}(x_i)\mathbf {v} - \mathbf {y} \right\| _2 + \lambda \left\| \mathbf {v} \right\| _1 \end{aligned}$$

where $\lambda$ is a regularization parameter, commonly estimated by cross-validation (when being defined over a grid), or in a faster, direct way through regularization choice methods (Bauer and Lukas 2011).

The LARS algorithm can be applied to achieve the model selection considering a delivered value of $\lambda$ simply a portion (or none) of the regressors possess corresponding nonzero weights. If $\lambda$ = 0, the problem converts unrestricted regression, and all weights are nonzero. As $\lambda _{max}$ grows from 0 to a provided value $\lambda _{max}$, the quantity of nonzero weights reduces to zero. For the problem considered in this paper, the $z_l$ regressors are the outputs of the meaningful neurons comprising the activation matrix $\mathbf {z}$ as columns. Thus, the LARS algorithm can be used to decide an optimal subset of the significant neurons that minimize (26) for a given value of $\lambda$. In order to increase statistical significance and robustness of the selection, we perform LARS on several bootstrap replications ($b_t$ in total) and select those neurons whose weights are non-equal to zero in at least $X\%$ of the regressors (with *X* typically a high value 90 to 95 in order to achieve statistical significance subject to a small (wrong rejection) level); *X* is termed as the consensus threshold.

The training model has synthesized as demonstrated in Algorithm 1. It has three parameters:

1. Initial Configuration: IBFC Fuzzification^2^
2. the number of bootstrap replications,*bt*;
3. the consensus threshold, *X*.

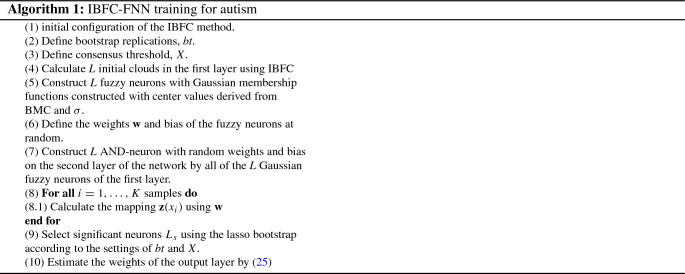


**Fig. 4:**
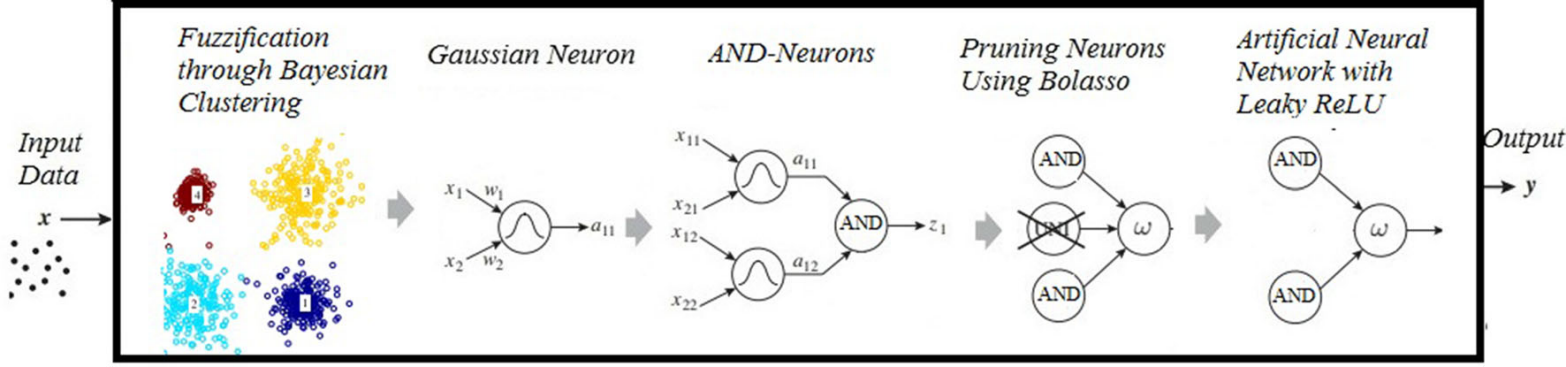
Block representation of the IBFC-FNN

The model’s main innovations are related to the frequent use of techniques based on the probability of problem data in concomitance with the extraction of fuzzy rules through simple processes (with fewer attributes to be chosen during the training process). Another highlight is the use of an activation function in the artificial neural network that improves the sensitivity of neuron responses, thus generating a more assertive defuzzification process. Figure 4 presents a block representation of the algorithm proposed in this paper.

## Test of classification binary patterns.

### Assumptions and initial test configurations

The test performed in this paper will act on the prediction of the autistic spectrum in children, adolescents, and adults. The tests will occur with the data obtained by the mobile application created by Thabtah (2017a) and made available in a vast database for machine learning problems called the UCI Machine Learning Repository.

To perform the tests, they will be performed in test blocks using a specific base and algorithms commonly used to classify binary patterns. For this, the general configurations of the tests are as follows:

- Data division: 70% for training and 30% for tests in all tests.
- Thirty repetitions of each test are performed to collect the means and standard deviations of each model.
- Randomized data.
- Hyperparameters of all models involved in the test were carried out through cross-validation (70–30).
- Outputs of the model were normalized to the interval [$-1$,1].
- All samples involved in the test were normalized with mean zero and variance one.
- The standard deviation in parentheses accompanies all the results presented in the tables.
- All algorithm processing time results are computed by the sum of the training and test times and expressed in seconds.

The factors evaluated in this paper are as follows:

27 $$\begin{aligned} \mathrm{acc}= & {} \frac{\mathrm{TP}+\mathrm{TN}}{\mathrm{TP}+\mathrm{FN}+\mathrm{TN}+\mathrm{FP}} \end{aligned}$$

28 $$\begin{aligned} \mathrm{AUC}= & {} \frac{1}{2}(\mathrm{sens}+\mathrm{spec}) \end{aligned}$$

In this context, consider specificity and sensitivity as:

29 $$\begin{aligned} \mathrm{sens} = {\frac{\hbox {TP}}{\hbox {TP}+\hbox {FN}}} \end{aligned}$$

30 $$\begin{aligned} \mathrm{spec} = {\frac{\hbox {TN}}{\hbox {TN} + \hbox {FP}}} \end{aligned}$$

where TP = true positive, TN = true negative, FN = false negative and FP = false positive.

The models used in all tests are presented in the itemization below. The fuzzy neural network models and the support vector machine use Matlab to perform their functions. The other models are available through a Java tool called WEKA (Hall et al. 2009).

- **BFNNRELU**: Bayesian fuzzy neural network proposed in this paper.^3^
- **ANFAND**: Fuzzy neural network proposed in De Campos Souza et al. (2018) that uses unineurons created in Lemos et al. (2010) and ANFIS proposed by Jang (1993) to fuzzification the training data.^4^
- **SVM**: The purpose of the support vector machine algorithm (proposed by Vapnik (2013)) is to find a hyperplane in an *N*-dimensional space (*N*—the number of features) that distinctly classifies the data points. It is an algorithm that can act with the classification of binary patterns with the focus on finding a margin that maximizes the separation between the classes in the hyperplane.^5^
- **MLP**: Multilayer Perceptron (McClelland et al. 1987) is one of the most widely used models in classifying binary patterns. It uses training based on the backpropagation technique and has a hidden layer.^6^
- **NB**: The Naive Bayes algorithm (John and Langley 1995) is a probabilistic classifier based on the Bayes theorem. He completely disregards the correlation between the variables.^7^
- **C4.5**: Generating a pruned or unpruned C4.5 decision tree (Quinlan 2014).^8^
- **RNT**: Random Tree (Aldous 1991) is used for constructing a tree that considers *K* randomly chosen attributes at each node.^9^

**Fig. 5:**
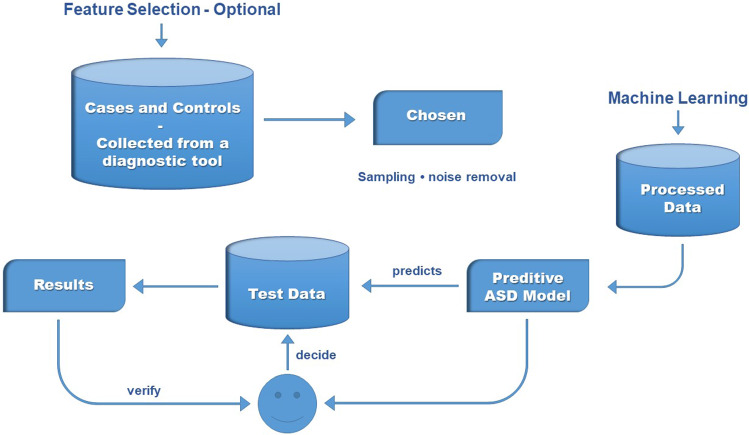
Diagram of the decision system on autism (Thabtah 2017b)

**Fig. 6:**
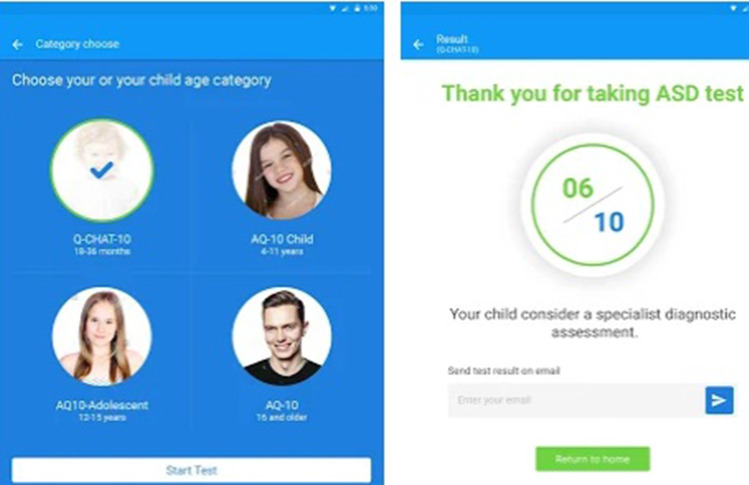
Mobile device interface for ASD identification (Thabtah 2017a)

The tests were performed on a computer with the following settings: Core (TM) 2 Duo CPU, 2.27 GHz with 3-GB RAM.

### Autism spectrum disorder tests app- dataset

Thabtah developed a mobile device program (Thabtah 2017a) to assist in application-directed inquiries whether or not a person is likely to possess autistic characteristics. In these circumstances, the software is downloaded free of cost from the mobile application stores, and subsequent choosing the language that the user will communicate with the software, a person begins to answer questions that were developed in studies of Thabtah (2017b) to support in the diagnosis of diseases. In Fig. 5, it can see whence the software operates and how it is supported by the people who respond to it all over the world. In Fig. 6, the interface presented to the user, the question styles, and the feedback received is highlighted.

This type of device was inspired by the experience of detecting autistic characteristics so that questions could help build tips for people who are not knowledgeable about the subject. This type of technology allows simple questions to be answered to explain early reports about the possibility of a person having autism.

This mobile application can be downloaded from any virtual store for mobile devices with access to an internet connection. Their questions involve personal and common characteristics, as well as perceptions about situations presented. It is worth mentioning that the doctor should give a conclusive diagnosis, but this tool can assist, for example, parents who have several doubts about the behavior of their children to begin the search for a specialist doctor.

**Fig. 7:**
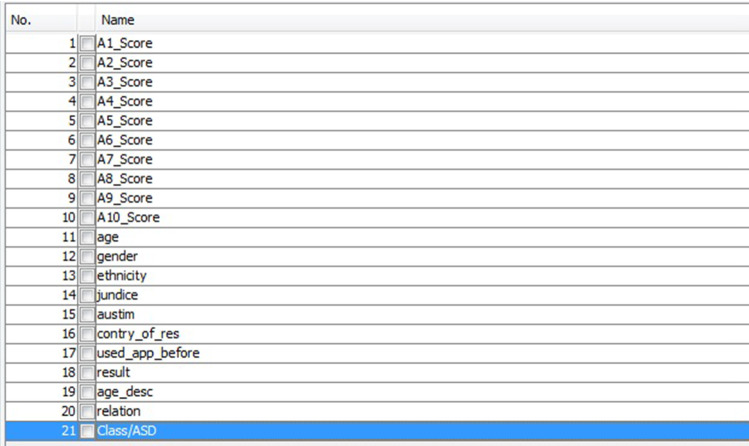
Relationship between autism and the age of children in the test

**Fig. 8:**
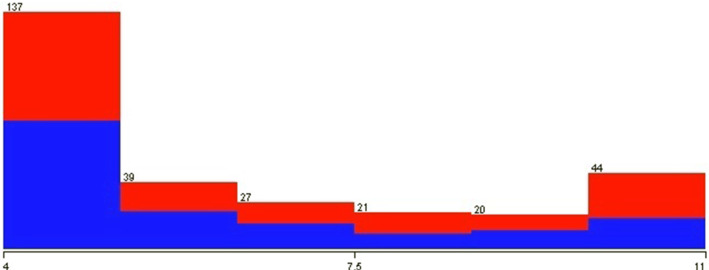
Relationship between autism and the age of children in the test

The database used in this test was provided by data collection in mobile applications after performing their activities according to the responses provided in the app. This data set is constructed through responses about situations raised by subject matter experts, besides age (which defines which group the data will be analyzed), citizenship, nationality, skin color, and family history of the disease. Textual values were transformed into sequential numbers for use in the model (based on their economic relevance in the world) and yes, and no values were converted to binary values. Finally, each subgroup (adults, children, and adolescents) is arbitrated according to an age range, as suggested by the dataset authors. The principal feature is age, gender, ethnicity, born with jaundice, the family member with PDD, country, and ten questions. All values used in the tests were collected by the mobile application proposed in Thabtah (2018), where there is no direct participation of people in this paper, and consequently, none of the data have the identification of the adolescent submitted to the test. For more information on the collected database, see Thabtah (2018).^10^,^11^,^12^

All records that had incomplete data were excluded from the assessment database. All dimensions collected in tests on mobile devices are shown in Fig. 7. The only dimension not considered in the evaluations is dimension 19 that was used to separate data from children, adolescents, and adults.

**Fig. 9:**
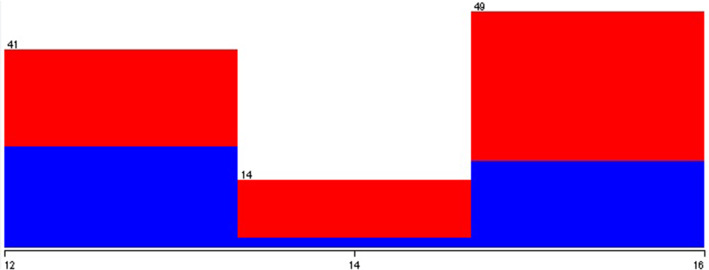
Relationship between autism and the age of adolescent in the test

**Fig. 10:**
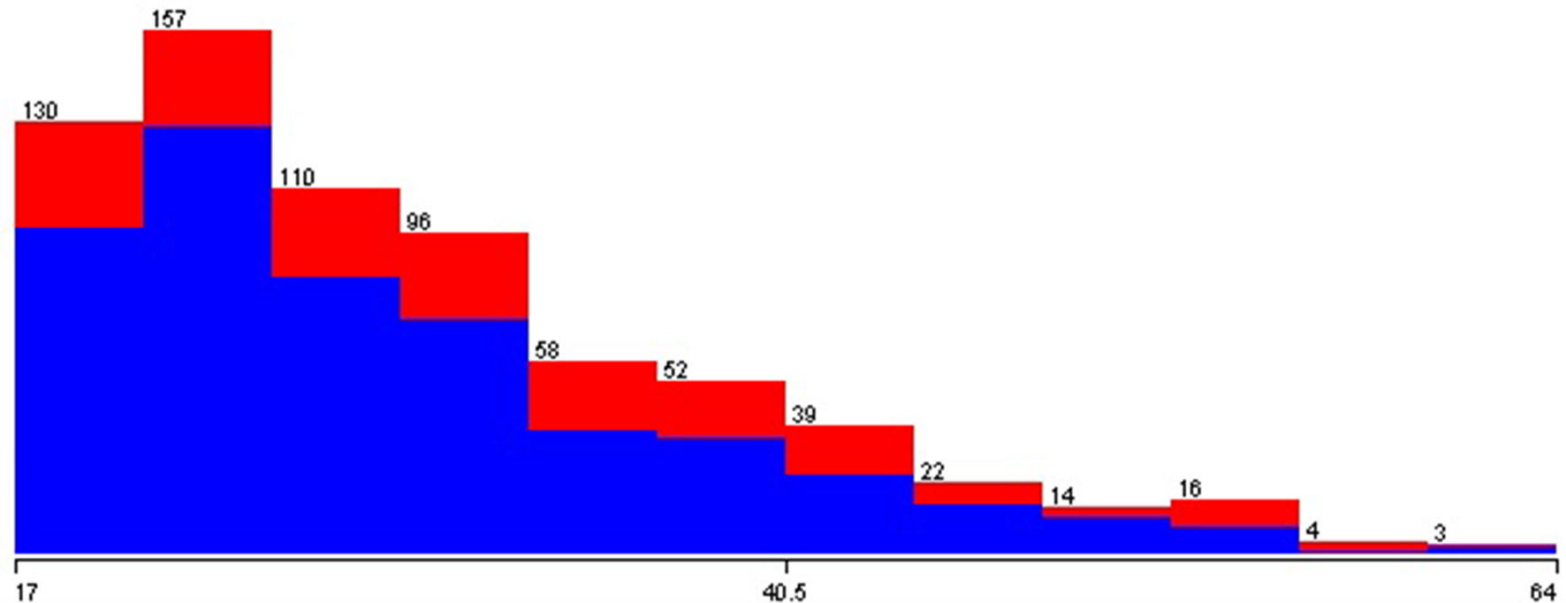
Relationship between autism and the age of adult in the test

What differentiates the database is how the author has grouped the samples obtained by the application according to the age collected. Figures 8, 9, and 10 show that the dispersion of ages and the blue and red colors represent people without and who has autism, respectively. It can be noted from these data that the most significant cases of autism present in the database are with younger people.

Table 1 presents the data of training, test, and identification of people with and without autism.

**Table 1:** Data set used in the experiments

Dataset	Autist.	Non-autist	Train	Test
Children	141	151	204	88
Adolescent	63	41	73	31
Adult	189	512	491	210

**Fig. 11:**
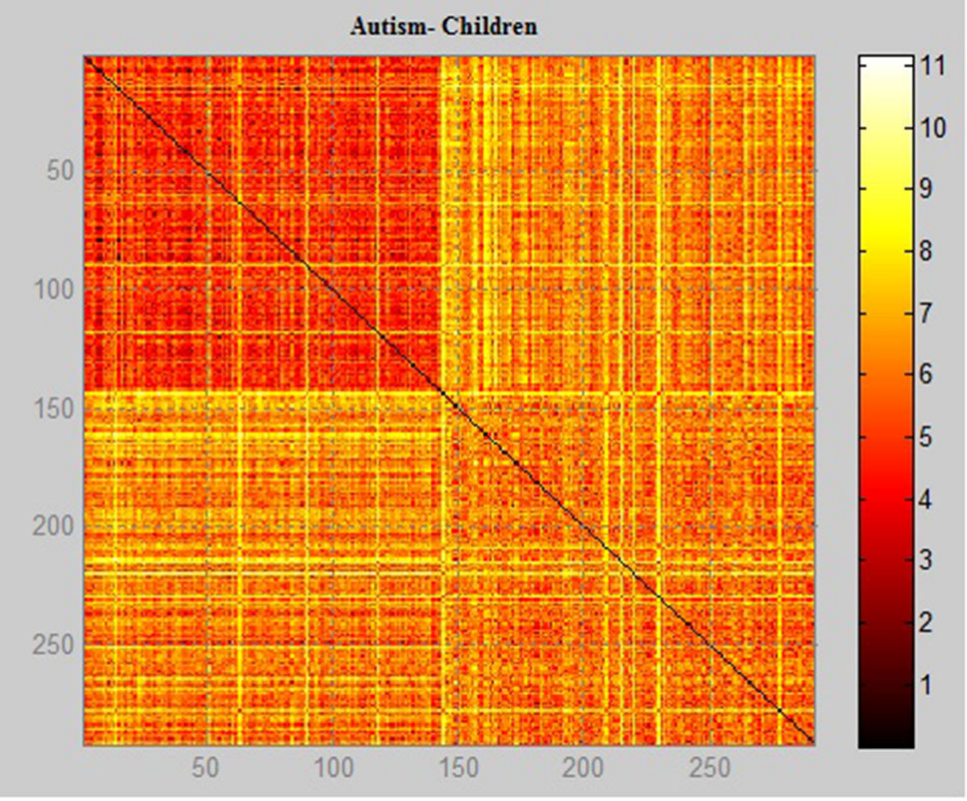
Graphical relationship—Euclidean distance of the autism database in children

**Fig. 12:**
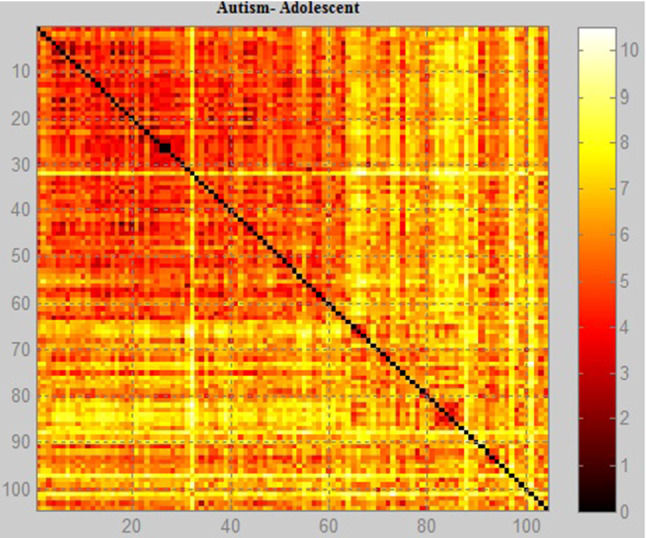
Graphical relationship—Euclidean distance of the autism database in adolescent

**Fig. 13:**
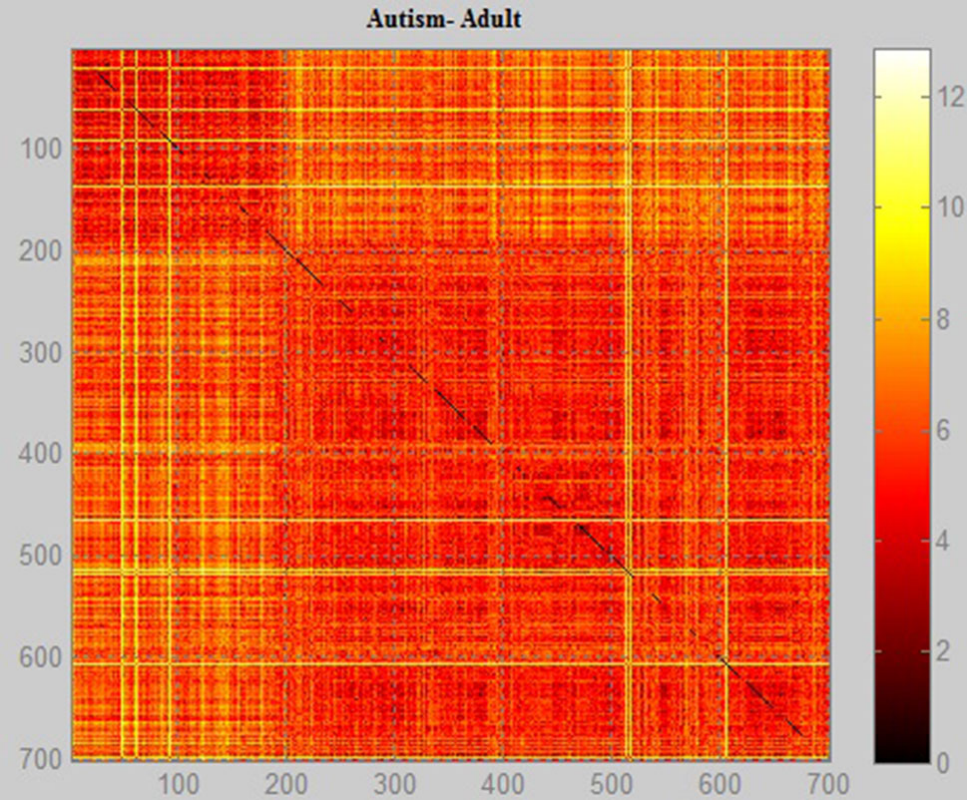
Graphical relationship—Euclidean distance of the autism database in adult

Figures 11, 12, 13 show the scale data and display the image using Euclidean distance weight function with the positive and negative data of the diagnoses of autism. It is possible to notice the difference in concentration of the data and the people difference diagnosed with the autistic spectrum in the research. The method used to obtain the image shows the problem data as an image so that it is possible to identify the concentration of people with autism and how often they appear in the database. Each element of the autism problem data matrix specifies the 1 pixel color of the image. The resulting image is an m-by-n pixel grid, where m is the number of columns, and n is the number of rows in the data matrix. The row and column indices of the elements determine the centers of the corresponding pixels.

### Autism pattern classification

**Fig. 14:**
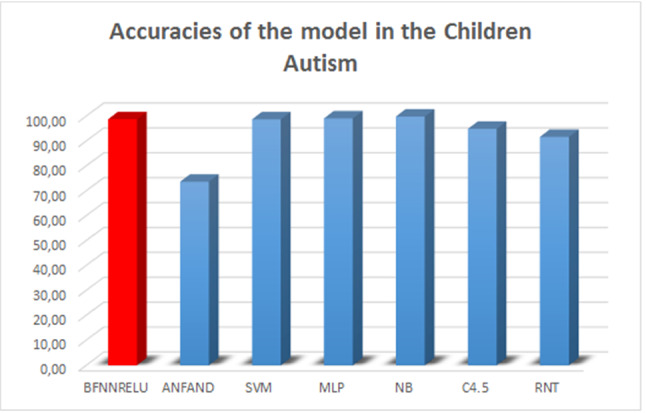
Graphical comparison of the results of the autism experiments in children

Next, the results obtained by intelligent models in the autism identification tests will be presented. Consider in the tables the bold values that obtained best results during tests and those highlighted with the symbol ’*’ as equivalent according to statistical tests when being compared to the result of the highest value (in bold) for the analyzed criterion. So, all values not highlighted with an asterisk indicate significantly lower performance. Table 2 presents the results of autism for children, and a graphical view of the results obtained in the experiments is presented in Fig. 14.

**Table 2:** Accuracies of the model in the children autism

Model	Acc.	AUC	Sens.	Spec.	Time (s)
BFNNRELU	98.73 (2.51)*	0.9697 (0.0402)*	0.9716 (0.0015)*	0.9678 (0.0212)*	45.18 (0.72)
ANFAND	73.63 (6.83)	0.6577 (0.0401)	0.5236 (0.2985)	0.7333 (0.4497)	53.26 (1.22)
SVM	98.67 (1.65)*	0.9913 (0.0012)*	0.9954 (0.0054)*	0.9872 (0.0014)*	190.16 (43.32)
MLP	99.05 (1.20)*	0.9906 (0.0021)*	0.9860 (0.0001)*	0.9952 (0.0017)*	187.51 (22.16)
NB	**99.82 (0.25)**	**1.0000 (0.0000)**	**1.0000 (0.0000)**	**1.000 (0.0000)**	16.21 (0.32)
C4.5	94.86 (4.06)	0.8920 (0.0014)	0.9987 (0.0040)*	0.7854 (0.0025)	0.32 (0.01)*
RNT	91.61 (8.51)	0.9259 (0.0002)	0.9210 (0.0012)	0.9108 (0.0007)	**0.29 (0.00)**

Bold values represent the best values in the test

In the obtained results, we highlight the models that use the concept of probability (NB) and support vector machine (SVM). It should be noted that the architecture proposed for the hybrid model significantly improved the accuracy of fuzzy neural networks and decreased the time to obtain these results (compare BFNNRELU and ANFAND). Furthermore, the proposed model (BFNNRELU) and the MLP, SMV, and NB models are statistically equivalent to a 95% confidence level for autism classification in children according to the paired t-test. On the other hand, the related machine learning models that obtained equivalent results to our approach are not interpretable, whereas our model offers linguistically readable rules, see Sect. 4.4.

**Fig. 15:**
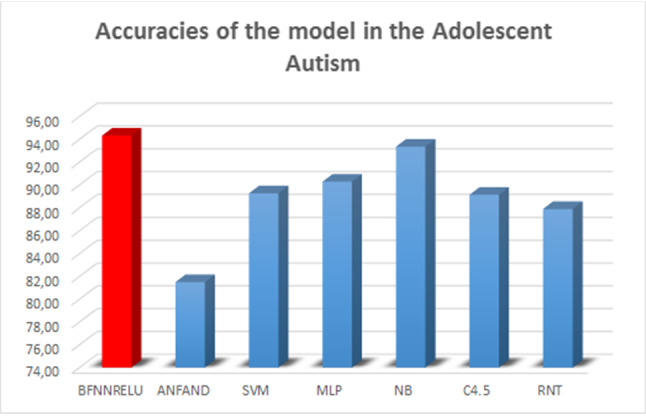
Graphical comparison of the results of the autism experiments in adolescent

Table 3 shows the tests for adolescents, and Fig. 15 shows the graphical results.

According to Table 3, it was found that the model proposed in this paper obtained the best accuracy results with a much shorter time than traditional models of the literature compared with the fuzzy neural network using the ANFIS in the input space partitioning (ANFAND). This demonstrates that knowledge about the characteristics of autism in adolescents can be more clearly evidenced by the model, thus making the knowledge extracted from the database more reliable.

Finally, Table 4 and Fig. 13 present the results obtained by the models in the classification of adults with autism.

**Table 3:** Accuracies of the model in the adolescent autism

Model	Acc.	AUC	Sens.	Spec.	Time (s)
BFNNRELU	**94.32 (6.14)**	**0.9475 (0.0614)**	**0.9571 (0.0902)**	0.9380 (0.0494)*	8.01 (1.17)
ANFAND	81.50 (9.46)	0.8061 (0.0900)	0.6758 (0.2142)	0.9259 (0.0764)	19.81 (0.32)
SVM	89.26 (4.65)	0.9098 (0.0071)	0.9452 (0.0054)*	0.8745 (0.0021)	150.16 (28.16)
MLP	90.28 (5.24)*	0.8926 (0.0018)*	0.8451 (0.0012)	0.9402 (0.0016)*	167.32 (48.09)
NB	93.35 (3.96)*	0.9459 (0.0013)*	0.9152 (0.0041)*	**0.9947 (0.0012)**	2.04 (0.02)
C4.5	89.13 (5.06)	0.8885 (0.0001)	0.8926 (0.0017)	0.8851 (0.0020)	0.21 (0.00)*
RNT	87.88 (13.63)	0.8638 (0.0025)	0.7963 (0.0102)	0.9313 (0.0042)	**0.05 (0.00)**

Bold values represent the best values in the test

**Table 4:** Accuracies of the model in the adult autism

Model	Acc.	AUC	Sens.	Spec.	Time (s)
BFNNRELU	97.28 (1.85)*	0.9559 (0.0400)*	0.9516 (0.0025)	0.9601 (0.0056)*	47.76 (3.15)
ANFAND	95.57 (1.35)*	0.9379 (0.0214)	0.9782 (0.0135)*	0.8976 (0.0417)	211.71 (2.43)
SVM	94.21 (0.76)	0.9350 (0.0012)	0.9754 (0.0025)	0.8946 (0.0017)	412.29 (53.19)
MLP	**99.91 (0.26)**	**0.9986 (0.0001)**	0.9995 (0.0001)*	**0.9977 (0.0001)**	167.32 (28.56)
NB	96.51 (1.03)*	0.9781 (0.0015)*	0.9715 (0.0041)*	0.9846 (0.0007)*	**0.35 (0.01)**
C4.5	91.86 (4.06)	0.8750 (0.0176)	**1.000 (0.0000)**	0.7500 (0.0041)	0.41 (0.02)*
RNT	95.30 (4.06)*	0.9382 (0.0098)	0.9700 (0.0048)*	0.9065 (0.0445)	0.79 (0.07)*

Bold values represent the best values in the test

**Fig. 16:**
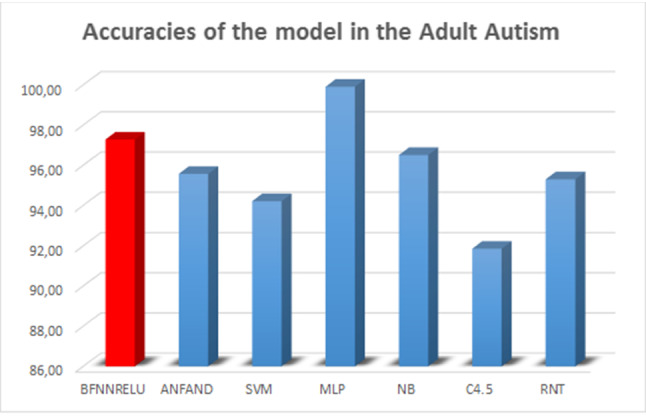
Graphical comparison of the results of the Autism experiments in adults

In the evaluation of autism in adults (Table 4), the model proposed in this paper was only numerically inferior to the MLP model, but its execution time was drastically lower; and again, as in the case of the children data set, from statistical point of view there was no real outperformance of our method (statistical equivalency based on a 95% confidence level). Although the MLP obtains the best accuracy and AUC results, the model is not capable of transmitting knowledge of the database. Another essential factor for the BFNNRELU model is the improvement of the results compared to an alternative fuzzy neural network model (ANFAND) (Fig. 16).

### Interpretability based on fuzzy rules extracted from the neurons

The main advantage of a hybrid system is its ability to extract accurate information from a database. Therefore, a fuzzy neural network model can identify characteristics that were not expected or are not identified when it acts efficiently within the resolution of the problem. This factor can actively aid other professionals’ training and disseminate more efficient practices to identify the symptoms of autism in people.

The following experiment presented a percentage of 90.91% accuracy, generating a total of 200 first fuzzy rules, which could be reduced to 17 neurons due to the selection strategy discussed above. The following two examples of fuzzy rules were used because their values have coherence in the identified centers and make the visualization simpler in the paper.

Linguistic terms have been assigned to the fuzzy sets found through Bayesian clustering to gain interpretability of the resulting rules. It may be noted that there are everyday situations among people who have or do not have autism.

**Rule 1:** If A1 Score is NO AND A2 Score is NO AND A3 Score is NO AND A4 Score is NO, AND A5 Score is YES AND A6 Score is YES AND A7 Score is YES AND A8 Score is NO AND A9 Score is NO AND A10 Score is NO AND age is VERY YOUNG AND gender is FEMALE AND ethnicity is WRITE-EUROPEAN AND jaundice is NO AND autism in the family is NO AND country is the UNITED KINGDOM AND results of the app is WEAK NO AND use the app before is NO AND relation is SELF the result is **children have autism.**

**Rule 2:** If A1 Score is NO A2 Score is YES AND A3 Score is YES AND A4 Score is YES AND A5 Score is YES AND A6 Score is YES AND A7 Score is YES AND A8 Score is NO AND A9 Score is NO AND A10 Score is YES AND age is VERY YOUNG AND gender is FEMALE AND ethnicity is WRITE-EUROPEAN AND jaundice is YES AND autism in the family is NO AND country is SPAIN AND results of the app is WEAK NO AND use the app before is YES, AND relation is SELF the result is that **children does not have autism.**

## Conclusion

The tests performed with the model proposed in this paper were able to extract knowledge from a complex database and maintain the ability to identify children, adults, and adolescents with autism correctly. The models used in the comparison prove that the answers are viable and reliable.

The use of fuzzy neural networks allows the creation of dynamic, intelligent systems, which can act in the construction of computational resources that facilitate the identification of diagnoses of people with autism syndrome, allowing the diagnosis to be more fully concluded, reducing the impacts on the lives of people with such a disease and at the same time creating for health professionals new forms of aid for more accurate diagnoses.

This work addressed a new clustering technique for the fuzzification process of fuzzy neural networks based on a Bayesian approach, streamlining the execution time of the algorithms, and improving the predictive capacity of the model. This factor generates a more compact and assertive set of neurons and consequently, fuzzy rules (being extracted from these neurons) to aid in the dynamics of autism diagnoses in people. A new selection technique combining lasso and boot-strapping to obtain the most informative neurons to explain the target also helped increase the compactness of the final rule base. The limitations of the work proposed in this paper are linked to fuzzy rules that can be redundant and to the execution time that can increase due to the number of dimensions of the problem or the exponential growth of data.

Studies on message enhancement and online identification of autistic characteristics through incremental rule-based and model adaptations may be relevant continuations of this paper. Other databases should emerge as new users access the app and answer their questions. Studies on autism in certain countries or at specific ages may also be conducted as a future extension of this paper.

## Footnotes

1 The original article dealt with problems with more complex values. In this context, we want to verify the variation of activation functions with smaller values closer to zero. Therefore, the focus of this paper is to use neurons with small changes in their inputs.

2 The same one used in: https://github.com/GatorSense/BayesianFuzzyClustering/blob/master/bfc/bfc_params.m.

3 *bt* = 16 and *X* = 0.5.

4 The value of *M* = 4, *bt* = 16 and *X* = 0.5.

5 In all tests that the model was used, one should consider the linear kernel. For more information on the code used, visit https://la.mathworks.com/help/stats/support-vector-machines-for-binary-classification.html.

6 In all tests, the following settings were used: batch size = 100, hidden layers = 1, learning rate = 0.3, momentum = 0.2, validation Threshold = 20.

7 In all tests, the following settings were used: batch size $=$ 100, use kernel Estimator = false, Supervised Discretization $=$ false.

8 In all tests, the following settings were used: batch size = 100, confidence factor = 0.25, min. num. obj = 2, num. Folds = 3, seed = 1, reduce Error pruning = false, unpruned = false, laplace = false.

9 In all tests the following settings were used: batch size = 100, breakTiesRandomly = false, KValue = 0, num. Folds = 3, seed = 1, minNum = 1.0, allowUnclassifiedInstances = false, maxDepth = 0.

10 To access the database for children, go to the following link: https://archive.ics.uci.edu/ml/datasets/Autistic+Spectrum+Disorder+Screening+Data+for+Children++ .

11 To access the database for adolescent, go to the following link:https://archive.ics.uci.edu/ml/datasets/Autistic+Spectrum+Disorder+Screening+Data+for+Adolescent+++.

12 To access the database for adult, go to the following link: https://archive.ics.uci.edu/ml/datasets/Autism+Screening+Adult.
